# Volatile aroma and physicochemical characteristics of freeze-dried coconut water using different encapsulating agents

**DOI:** 10.1038/s41598-023-46610-1

**Published:** 2023-11-17

**Authors:** Yardfon Tanongkankit, Sunee Eadmusik, Pakkawat Detchewa, Tanakwan Budsabun, Wattana Panphut, Nattakan Jakkranuhwat, Sriwiang Rittisak, Panadda Nonthanum, Chanthima Phungamngoen

**Affiliations:** 1 Department of Food Technology, Faculty of Technology, Khon Kaen University, Khon Kaen, Thailand; 2 Department of Agricultural and Food Engineering, Faculty of Engineering and Agro-Industry, Maejo University, Chiang Mai, Thailand; 3 Department of Agro-Industry and Management, Faculty of Agro-Industry, King Mongkut’s University of Technology North Bangkok, Prachinburi, Thailand; 4 Department of Innovation and Product Development Technology, Faculty of Agro-Industry, King Mongkut’s University of Technology North Bangkok, Prachinburi, Thailand; 5 Department of Industrial Microbiology Program, Faculty of Science and Technology, Suan Sunandha Rajabhat University, Bangkok, Thailand

**Keywords:** Engineering, Materials science

## Abstract

This research studied how different types and concentrations of encapsulating agents impacted freeze-dried coconut water products. Volatile aroma and physicochemical product characteristics were evaluated. The encapsulating agents were maltodextrin 4–8% (w/v), polydextrose 4–8% (w/v) and xanthan gum 0.1–0.3% (w/v). A plate freezer and an air blast freezer were used to pre-freeze the coconut water before drying. Freezing time had no impact on moisture content and water activity. The flavor compounds of coconut water is composed of alkanes, aldehyde, ketones, organic acids and some other flavor substances. Encapsulating agents are the main factors affecting the flavor of coconut water. Optimal conditions for producing dried coconut water were adding polydextrose at a concentration of 8%. Volatile compounds were assessed under different conditions of SPME- GC-TOFMS. The composition of flavor compounds in coconut water is complex and mainly includes esters, aldehydes, and phenols. Results showed that encapsulating agents improved the volatile aroma of dried coconut water products.

## Introduction

Coconut water has recently gained popularity among consumers as a healthy sports drink with rehydration potential^1^. However, limited product shelf life and transportation difficulties are stumbling blocks for wider usage. The catalytic activities of polyphenol oxidases (PPO) and peroxidases (POD) reduce the public appeal of coconut water during processing. Thermal processing methods are commonly employed to preserve coconut water but these treatments diminish overall product acceptance with detrimental effects on color, clarity, and sensory properties.

Freeze drying maintains the typical properties of raw materials, with nutrient content and taste of the powdered freeze-dried product similar to fresh coconut water^2^. The freeze drying process involves addition of filler or components to increase the stability of the final product^3^. Boonnumma et al.^4^ reported that freeze-dried products must maintain the stability of rehydration power and material structure. Active compounds such as L-arginine, magnesium (Mg), and potassium (K) in a powder made from young coconut water processed by freeze drying and fortified with vitamin E 400 IU were higher than the product obtained through the spray-drying method. Flavor is one of the most important factors that determines the quality and acceptability of coconut water. The composition of flavor compounds in coconut water is complex and mainly includes esters, aldehydes, and phenols. Therefore, it is particularly important to study the odor activity values of flavor compounds as novel food research and development^5,6^.

Powdered coconut drinks show reduced levels of aroma, flavor, and sweetness compared to fresh samples because water and sugar product levels are lowered by freeze drying^7^. Polysaccharide gums derived from plants are generally used as carrier materials to encapsulate vitamins, minerals and colorants^8^ and improve powder yield^9,10^. Exudate gums have been valuable international trade items in the food, pharmaceutical, and other industries for thousands of years^11^. During the encapsulation process of fruit juices, hydrogen bonding and dipole–dipole interactions occur between natural gums as wall materials and phenol compounds, mainly due to the presence of free hydroxyl groups in the juice and also in the gum^12^. Nambiar et al.^13^ reported that higher *Moringa oleifera* gum (MG) concentration and lower maltodextrin (MD) concentration significantly improved encapsulation efficiency. Encapsulation efficiency was enhanced because of the presence of hydrophilic hydroxyl groups in the MG polysaccharide structure which interacted with hydrophilic groups in young coconut water.

Copious research has investigated the qualities of microencapsulated fruit juices. However, no published papers have compared the volatile aroma of powdered coconut water obtained by freeze drying using different encapsulating agents. This study assessed the merits of diverse encapsulating agents on the volatile aroma physicochemical characteristics of freeze-dried coconut water products.

## Materials and methods

### Sample preparation

Fresh coconuts were purchased from a local market in Prachinburi Province, Thailand. The coconut shell was opened to obtain fresh coconut water. Sugar was added to sweeten to 18°Brix with 0.4% (w/v) salt.

### Encapsulating agents

In this study, the three encapsulation agents were maltodextrin (MD) at 4, 6 and 8% (w/v), polydextrose (PD) at 4, 6 and 8% (w/v) and xanthan gum (XG) at 0.1, 0.2 and 0.3% (w/v). The naturally contaminated microorganisms in coconut water were reduced by heating the samples at 75 °C for 30 s. After heating, the sample was immediately cooled in a water bath at 5 °C.

### Freeze drying

Ten grams of coconut water were poured into a silicon mold 6 × 6 × 2.5 cm. Two different freezing methods were studied using a plate freezer at − 40 °C and an air blast freezer at − 30 °C for 24 h before freeze drying by a freeze dryer (ScanVac Model CoolSafe 4-15L, Germany) under 50 mPa pressure for 48 h.

### Isolation of volatile aroma compounds

Solvent extraction was performed following the modified method of Nasution et al.^14^. Five grams of the encapsulated powders were diluted with 250 mL distilled water. A coconut water sample (250 mL) was added with 60 g of NaCl and extracted with 250 mL dichloromethane using 30 µL of 2-Octanol (1.45 mg/mL; Sigma-Aldrich, USA) as the internal standard. Prior to chromatography analysis, it was concentrated to a final volume of 0.5 mL under gentle stream of N_2_ gas. Lastly, 1 µL of extract was injected by on-column mode to a gas chromatography-time of flight mass spectrometry (GC-TOFMS).

### Headspace–solid phase microextraction (HS-SPME)

The HS-SPME method was carried out on coconut water (5 mL) using a 50/30 µm divinylbenzene (DVB)/carboxen (CAR)/polydimethylsiloxane (PDMS) SPME fiber (Supelco, USA). The sample was placed in a 20 mL vial and added with 5 µL of 2-octanol (50 ng/mL) and 1 g NaCl. Next, the sample was equilibrated at 40 °C for 40 min in a water bath while stirring at low speed. Thermal desorption of volatile compounds from the fiber was carried out for 14 min in an SPME inlet liner (0.75 mm I.D.; Supelco, USA).

### Gas chromatography–time of flight mass spectrometry analysis of volatile aroma compounds

The samples were analyzed using a GC (7890A; Agilent Technologies, USA) equipped with a TOFMS (Pegasus 4D, LECO Corp., USA). Separation of compounds was performed on a polar Stabilwax® capillary column with a polyethylene glycol stationary phase and a non-polar Rxi5ms capillary column with a diphenyl dimethyl polysiloxane stationary phase, both with similar dimensions (30 m × 0.25 mm I.D. × 0.25 µm film thickness; Restek Corp., USA). The inlet port, transfer line, and M/S source were set at 250 °C, 250 °C, and 200 °C, respectively. Helium was used as the carrier gas at a flow rate of 1 mL/min. The oven temperature was programmed to start from 35 °C, held for 5 min, ramped at 4 °C/min to 240 °C, and held for 10 min. Other conditions were set as follows: scanning mass range 30–300 m/z with an acquisition rate of 2.74 scans/s and electron ionization energy 70 eV.

### Identification and quantification of volatile aroma compounds

Measured mass spectra were compared to the database, with volatile compounds identified by matching their retention indices (RI) calculated based on a series of n-alkanes (C6-C30 for vanillin and benzoic acid, C6-C22 for hentriacontane). Several selected coconut-related compounds were further identified after injection of standard compounds into a similar GC system. The developed calibration curve had a linear correlation coefficient of 0.97.

### Determination of physicochemical characteristics

#### Dried coconut water

Each sample (3–5 g) was assessed for moisture content using a standard gravimetric method (AOAC, 2000), with water activity (*a*_w_) determined using a water activity meter (Series 3TE, AquaLab, Washington).

#### Coconut water

Sample color was measured by a Hunter Colorimeter (Hunter Lab, Model Colorflex45/0, Virginia). Reflection spectra were registered and Hunter Lab color parameters for 10° vision angle and D65 illuminant were calculated. Total color change (Δ*E*) was calculated as:

1 $$\Delta E = \sqrt {\left( {L - L_{0} } \right)^{2} + \left( {a - a_{0} } \right)^{2} + \left( {b - b_{0} } \right)^{2} }$$

where *L*_0_, *a*_0_ and *b*_0_ are color values of the fresh coconut water sample.

A dried sample (6 g) was extracted with 300 mL of distilled water for 1 min. The apparent viscosity of the sample was determined at 25 °C using a Brookfield Viscometer, Model LVDV-II + Pro (Brookfield Laboratories, Massachusetts) at 100 rpm. All determinations were performed in triplicate, with results expressed as mean values.

### Experimental and statistical analysis

All experiments were carried out in triplicate, with mean average values and standard error (± SD) calculated. Significant differences between means were determined using the MINITAB package. All methods were carried out in accordance with relevant guidelines.

## Results and discussion

**Figure 1:**
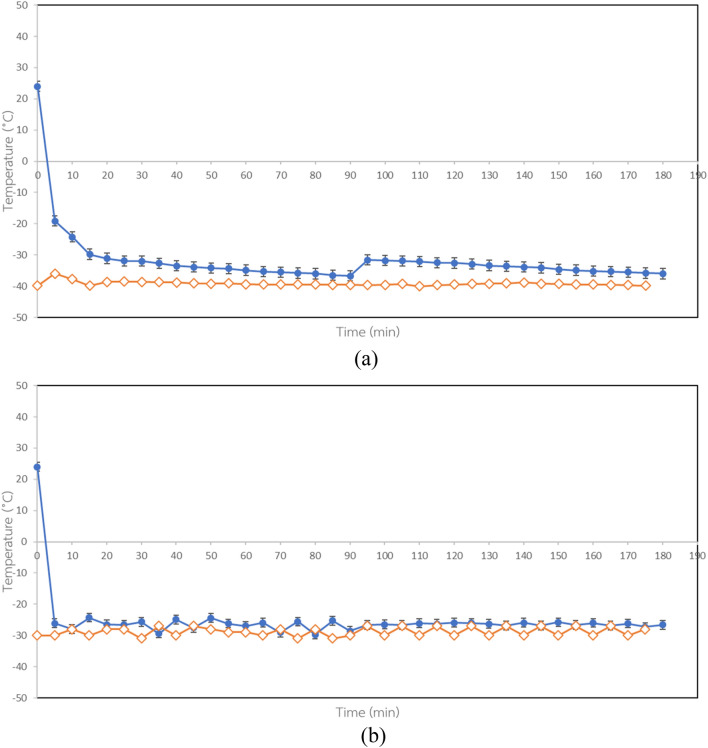
Temperature profiles of samples during the different freezing processes: (**a**) Plate freezer and (**b**) Air blast freezer. (blue circle) Coconut water sample (red diamond) Freezing temperature.

Figure 1 shows the temperature profiles of samples during the two freezing processes divided into three steps of cool down, freezing and crystallization. Thermocouples were placed at the cold slowest freezing point inside the samples. The temperature of the samples before freezing was 25 °C. Results showed that the plate freezer and air blast freezer exhibited similar behavior. Sample temperature decreased continuously as freezing time increased and approached the freezing temperature within 60 and 20 min for the plate freezer and air blast freezer, respectively. During the freezing process, heat transfer in the products was by conduction and convection^15^. During conduction, heat moves from one particle to another in a straight line, while convection heating is much more rapid, with coconut water heated more quickly than the pieces of sample in the container^16^. Air blast freezing involves using a lower freezing temperature with higher velocity of air circulation,therefore, freezing rates are higher than for plate freezing. The freezing time for the plate freezer sample was longer than the air blast freezer sample because air velocity flow in the air blast freezer was 6 m/s, while there was no air movement in the plate freezer.

### Moisture content and water activity of dried coconut water

**Figure 2:**
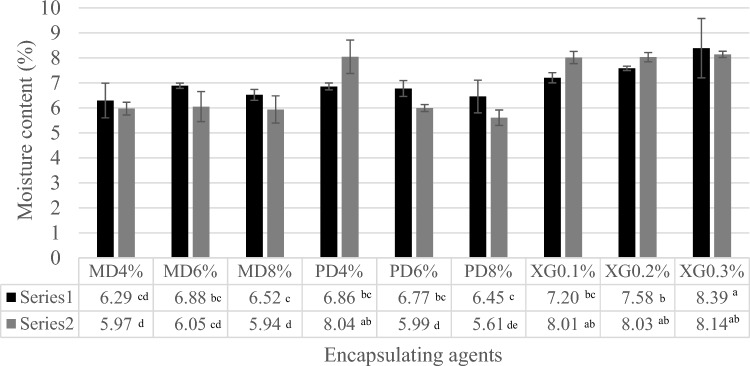
Moisture content of samples during the different freezing processes: (black square) Plate freezer and (gray square) Air blast freezer. MD = Maltodextrin, PD = Polydextrose and XG = Xanthan gum.

Moisture content of the dried samples was controlled below 10% wb. at all drying conditions, as shown in Fig. 2, with final moisture content 5.94–8.39% wb. Moisture content decreased as amounts of encapsulation agents increased because higher concentrations of encapsulation agents protected against moisture evaporation from food matrices. Plate freezing at different concentrations of MD and PD had no effect on moisture content, while adding XG in coconut water at higher concentrations impacted moisture content. XG is a hydrocolloid that absorbs water, thus affecting the dispersion and gelatinization of the product^17^.

**Figure 3:**
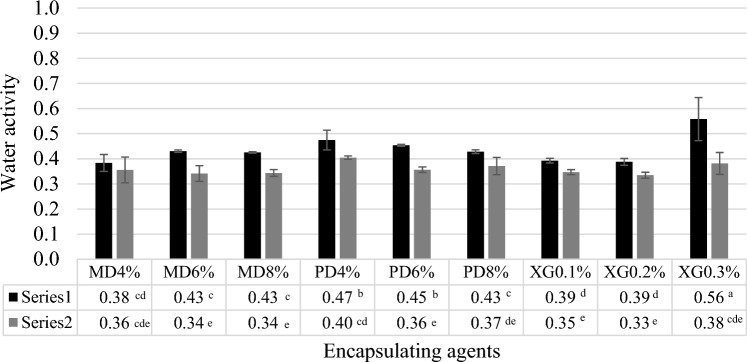
Water activity of samples during the different freezing processes (black square) Plate freezer and (gray square) Air blast freezer. MD = Maltodextrin, PD = Polydextrose and XG = Xanthan gum.

Water activity (*a*_w_) values of dried coconut water are shown in Fig. 3. The initial *a*_w_ of coconut water was 1.000. The dried samples showed a narrow range of 0.33–0.56 because the final moisture content was controlled. Water activities of the air blast freeze samples were lower than the plate freeze samples. The Thai Community Product Standard states that dried product should have an *a*_w_ less than 0.6. Results showed that the *a*_w_ of the dried products were all below this standard limit.

### Color

**Figure 4:**
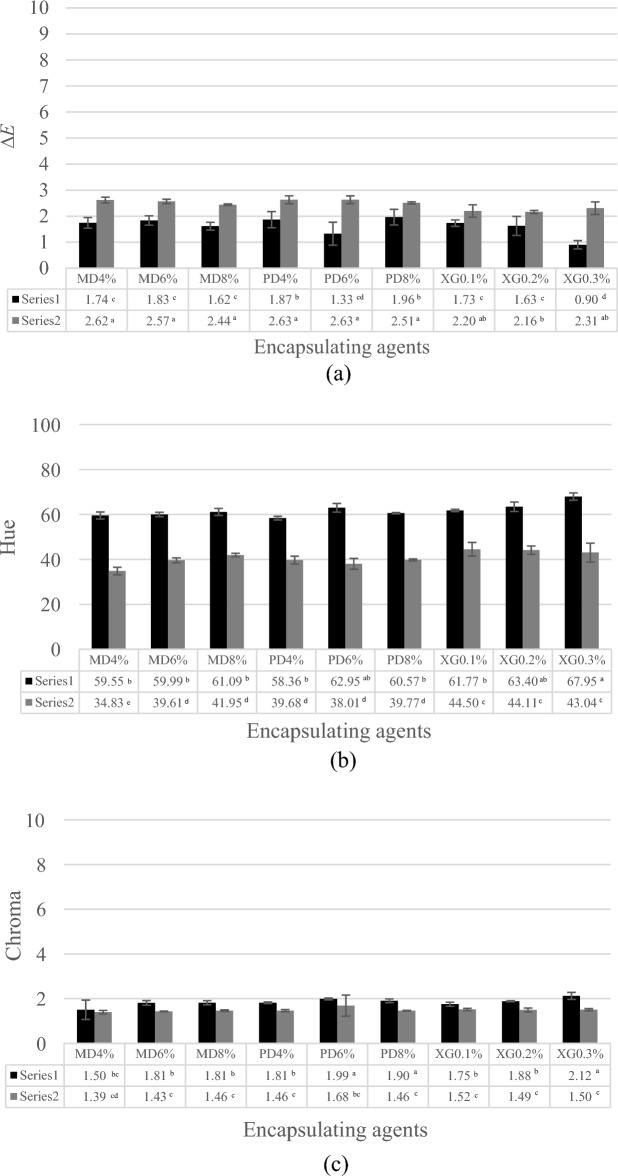
Δ*E* (**a**), hue (**b**) an chroma (**c**) of samples during the different freezing processes: (black square) Plate freezer and (gray square) Air blast freezer. MD = Maltodextrin, PD = Polydextrose and XG = Xanthan gum.

The L* values of both the plate freezer and air blast freezer were not significantly different, with L* b* a* values of all samples showing no clear trends. Total color difference (Δ*E*), hue and chroma are shown in Fig. 4a–c. Plate freezer samples showed that the hue added with all three encapsulating agents ranged from 65.05 to 77.87 indicating a yellow hue, while the air blast freezer samples showed hue ranging from 40.73 to 67.07. Adding encapsulating agents combined with plate freezing exhibited lower ∆*E* values than air blast freezing. Some moisture content was crystallized during plate freezing, resulting in the smallest crystallized ice with reduced exposure to thermal stress. In general, lowering the freezing temperature helped to prevent the browning reaction and product color degradation^18^. The principle of freeze drying technology starts with the freezing process and continues with drying by removing/separating most of the water in the material through the mechanism of sublimation. Therefore, processes of gelatinization, caramelization, and denaturation do not occur, thereby preventing a change in crust formation for the dry part of the food. The browning reaction is initiated by thermal effects. All samples were subjected to heating at 75 °C for 30 s. After applying heat pre-treatment, the natural enzyme population was reduced^19^. Chroma is associated with color intensity and represents saturation, while the hue angle value indicates how color is perceived. Chroma values of dried coconut water using plate freezing were 1.50 to 2.12. Plate freezing showed higher chroma values than samples subjected to air blast freezing, with chroma values ranging 1.39–1.68. Results indicated that chroma and hue values exhibited similar behavior for both freezing methods.

### Viscosity

**Figure 5:**
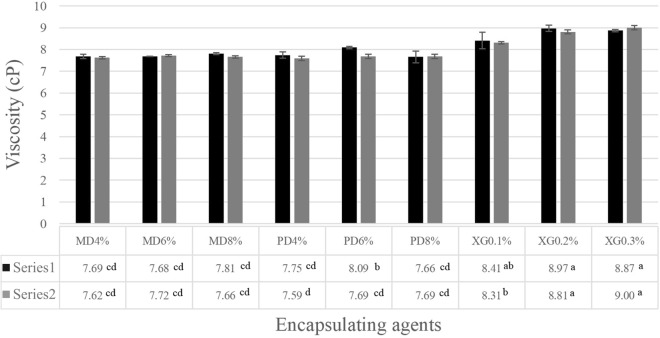
Viscosity of samples during the different freezing processes: (black square) Plate freezer and (gray square) Air blast freezer. MD = Maltodextrin, PD = Polydextrose and XG = Xanthan gum.

Figure 5 shows the viscosity of coconut water. All samples with encapsulation agents or sterilizers exhibited higher viscosity than fresh coconut water because the stabilizers maintained coconut water stability between the two immiscible liquids. Adding XG to the food system showed higher viscosity than adding MD and PD. An increase in the apparent viscosity of coconut water was observed with increasing XG concentration. This observation concurred with Fonseca et al.^20,21^ who reported that XG addition increased the viscosity of the continuous phase and improved emulsion stability. Adding higher concentrations of MD and PD had no significant effect on the viscosity of coconut water. PD has a similar sugar structure to MD, with slight increases in sample viscosity when added in larger amounts. MD addition caused the proportion of water in the product to decrease, with low concentration of MD giving low viscosity. The freezing process had no significant effect on the viscosity of coconut water.

Samples with high solubility and low ∆*E* values had similar viscosity to fresh coconut water and were suitable for production of dried coconut water. The recommendation condition was adding MD 8%, PD 8% and XG 0.1% to the samples respectively and using the plate freezing method before drying. These three conditions were selected to further study their effects on the volatile aroma compounds of coconut water.

### Volatile aroma compounds

Table 1 shows how the selected encapsulated agents impacted the volatile aroma compounds in coconut water. The flavor of coconut water is composed of alkanes, aldehyde, ketones, organic acids and some other flavor substances. Encapsulating agents are the main factors affecting the flavor of coconut water. Coconut water samples contain more polar groups such as organic acids, alcohols and phenols, and ketones. Extractable substances include 3-hydroxy-2-butanone and 2,3-butanediol, phenol, nonanal and ethyl tridecanoate. 3-Hydroxy-2-butanone is a ketone compound with a sweet aroma, while 2,3-butanediol are alcohol compounds. 2,3-butanediol has a characteristic smell of butter and cream. Hentriacontane has a sweet, grainy and caramelized appearance, while 4-hydroxy-3-methoxybenzaldehyde (Vanillin) emits the smell of popcorn, bread, and fragrant rice. Benzoic acid and nonadecane emit sweet, creamy coconut aromas^22^. Therefore, these compounds play important roles in contributing to the sweet odor and scents in fragrant coconut water. The results are close to those reported by Marchi et al.^1^, who found the identified 73 volatile compounds in the headspace of young green Thai coconuts (*Cocos nucifera* cv. Nam Hom) including alcohols, aldehydes, and ketones with smaller amounts of esters, lactones, terpenes, ethers, and heterocycles.

**Table 1:** Effect of selected encapsulated agents on volatile aroma compounds in coconut water.

Compound	Retention time (RT: min)	Retention index (RI)*	MD 8%	PD 8%	XG 0.1%
Amine					
2-Hexanamine	1.53	720			✓
2-pentanamine	1.60	ND		✓	
Benzeneethanamine	1.86	ND		✓	
1-octanamine	2.54	179			✓
2-pentanamine	2.72	ND			✓
Acid					
Formic acid	3.88	1510		✓	
Acetic acid	4.10	1267	✓	✓	✓
Isopropoxycarbamic acid	4.93	ND			✓
Benzoic acid	17.67	1159	✓	✓	✓
Phosphonic acid	10.98	ND		✓	✓
Oxalic acid	21.35	780		✓	✓
Butanoic acid	24.56	1676	✓	✓	
Decanoic acid	25.26	1373	✓	✓	✓
Dodecanoic acid	30.40	2508		✓	
Phthalic acid	38.65	1627	✓	✓	
Propanedioic acid	41.53	ND	✓	✓	
Alcohol					
Ethanol	1.65	925	✓	✓	✓
1-octanol	14.03	1555	✓	✓	✓
Cyclobutanol	53.28	ND			✓
Ester					
t-butyl ester	13.91	ND	✓	✓	✓
Ethyl ester	18.59	1269		✓	
Diethyl ester	16.95	ND	✓	✓	
Ethyl tridecanoate	31.29	1676			✓
Aldehyde					
Heptanal	8.00	1210		✓	
Hexanal	12.50	1083	✓	✓	✓
Nonanal	15.22	1382		✓	
Decanal	18.87	1506	✓	✓	
Isophthalaldehyde	19.66	2341		✓	
4-hydroxy-3-methoxybenzaldehyde					
(Vanillin)	27.31	1372	✓	✓	✓
Benzaldehyde	25.07	1543		✓	✓
Ketone					
3-hydroxy-2-butanone	6.31	1268	✓	✓	✓
Acetophenone	13.80	1650		✓	
Ethanone	23.37	1512		✓	✓
Propanone	46.80	850		✓	
Alkane					
Cyclopentane	16.40	700	✓	✓	
Dodecane	18.64	ND	✓		
Nonadecane	30.56	ND	✓	✓	✓
Octane	24.41	ND	✓	✓	
Tetradecane	25.36	ND	✓	✓	
Pentacosane	28.36	ND			✓
Hentriacontane	34.49	485	✓	✓	
Others					
2,3 butanediol	5.43	1522	✓	✓	✓
Phenol	28.89	1870			✓
dl-Alanine	1.82	ND		✓	

* Retention index (RI) were calculated based on a series of n-alkanes (C6-C30 for polar column) from NIST Standard Reference Database Number 69 (https://doi.org/10.18434/T4D303).

ND is not found in the database.

Table 2 shows volatile aroma compounds in coconut water with different conditions. The encapsulating agents exhibited different physical properties due to differences in their chemical structure and intermolecular force^23^. The encapsulates containing 4-hydroxy-3-methoxybenzaldehyde (Vanillin), benzoic acid, hentriacontane in the rehydrated coconut water product. These substances are frequently found as aromatic compound in fresh coconut water. In the case of the 8% MD and 8% PD encapsulate, freeze drying process induced transition could still be observed, as opposed to 0.1% XD encapsulates. Normally aldehyde and ketone also changed in concentration due to heat treatment during the encapsulation process. A higher number of ketones post-processing was hypothesized as a result of the Maillard reaction, while reduction of ketones occurred from oxidation or thermal degradation. Moreover, various probiotic strains with non-toxicity and strong biocompatibility form gels during encapsulation using MD^24^. MD is a popular easily available coating material because it is non-toxic with low cost, good solubility and has low viscosity even at high solid content. MD are partial starch hydrolysis products with dextrose-equivalent values below 20. Their ability to reduce the hygroscopicity and stickiness of dried powders makes them a popular option for encapsulating fruit juices that tend to become adhesive upon drying due to their high sugar and organic acid content^25^ However, certain limitations exist regarding encapsulation capacity at higher aromatic compound concentrations.

**Table 2:** Volatile aroma compounds in coconut water with different conditions (ug/L).

Compound	Fresh coconut water	8% MD	8% PD	0.1% XG
4-hydroxy-3-methoxybenzaldehyde (Vanillin)	2351.88 ± 296.57^a^	321.66 ± 32.15^c^	611.52 ± 164.42^b^	11.18 ± 3.21^d^
Benzoic acid	336.41 ± 88.96^a^	132.69 ± 17.58^b^	162.29 ± 21.96^b^	5.34 ± 0.47^c^
Hentriacontane	57.22 ± 15.11^a^	50.97 ± 4.01^a^	53.93 ± 14.92^a^	ND

Different letters in the same line represent significant differences (*p* < 0.05).

ND is not detected.

Polydextrose (PD) is a polysaccharide, with an average degree of polymerization of 10 glucose residues, obtained by thermal polymerization of D-glucose in the presence of sorbitol and phosphoric acid. PD is mainly used as a sugar substituent and as dietary fiber in foods and can provide physiological effects similar to dietary fibers^26^. This polysaccharide has been studied as a wall material for the encapsulation of probiotics^27,28^, and shows potential as a microencapsulation agent. Moreover, PD has demonstrated retention of volatile aroma compounds in coconut water, with physicochemical characteristics similar to MD. For example, the retention of 4-hydroxy-3-methoxybenzaldehyde (vanillin) by 8% MD, 8% PD and 0.1% XD were 321.66 ± 32.15, 611.52 ± 164.42, 11.18 ± 3.21 ug/L, respectively. The retention of vanillin in fresh coconut water was 2351.88 ± 296.57 ug/L.

Although XG is commonly used as wall material in the encapsulating process. For example, Ortega et al.^29^ found that addition of XG decreased water solubility but increased the encapsulation efficiency of clove oil in microcapsules. Release studies indicated that the presence of XG decreased the release rate of clove oil from microcapsules. Nambiar et al.^13^ found that total phenol content and radical scavenging activity of coconut water were relatively low compared to XG, whereas MD had no phenol content and antioxidant activity. MG concentration showed a significantly positive linear correlation with DPPH and ABTS· + radical scavenging activity of spray-dried powder. The high antioxidant activity of XG was attributed to the polyphenol compound leucoanthocyanin which enhanced the antioxidant activity of the encapsulated powder. In the cases of 8% PD and 8% MD, the retention of vanillin, benzoic acid and hentriacontane were significantly higher than 0.1% XG. This could suggest that, in the case of 8% PD, the encapsulation capacity of the wall material exhibited the highest all volatile aroma compounds retention, and therefore, it could represent the best choice for designing stable encapsulate formulations for freeze dried coconut water.

## Conclusions

Factors affecting the volatile aroma and coconut water properties after freeze drying were investigated. Results showed that higher concentrations of encapsulation agents reduced moisture evaporation from food matrices. MD and PD with plate freezing at different concentrations had no effect on moisture content and water activity. Types and concentrations of encapsulating agents significantly impacted viscosity and color of freeze-dried products after dissolution. The optimal condition was coconut water with 8% PD. The composition of flavor compounds in coconut water is complex and mainly includes esters, aldehydes, and phenols. Results showed that encapsulating agents improved the volatile aroma of dried coconut water products.

## Data Availability

The datasets used and/or analyzed during the current study available from the corresponding author on reasonable request.
